# Motor Imagery Practice and Cognitive Processes

**DOI:** 10.3389/fpsyg.2020.00394

**Published:** 2020-03-03

**Authors:** Aidan Moran, Helen O'Shea

**Affiliations:** School of Psychology, University College Dublin, Dublin, Ireland

**Keywords:** motor imagery practice, mental imagery, cognitive processes, motor simulation theory, working memory

## Introduction

Mental imagery is a multimodal cognitive simulation process that enables us to represent perceptual information in our minds in the absence of actual sensory input (Munzert et al., 2009). Within this construct, motor imagery (MI) is a dynamic mental state during which the representation of a given motor movement is rehearsed in working memory without overt motor output (Decety, 1996). A popular and widely investigated application of MI is “motor imagery practice” (MIP; also known as “mental practice”) which is a mental simulation process that involves the systematic use of imagery to covertly rehearse a movement without actually executing it (Di Rienzo et al., 2016). Research shows that MIP is effective in enhancing skilled performance both in healthy populations (Driskell et al., 1994) and in clinical groups (e.g., Mateo et al., 2015). It not only improves motor learning (e.g., Kraeutner et al., 2016) but also induces “neural plasticity” (e.g., Debarnot et al., 2014) or the capacity of the brain to reshape its physical structure as a direct result of repeated experience.

Despite such findings, relatively little is known about certain cognitive changes induced by, and/or associated with, MIP. In this opinion piece, we consider four such questions. Firstly, how do participants in MIP studies construct procedural representations from the instructions presented to them in imagery scripts? Secondly, what is known about the interaction between working memory (WM) and MIP? Thirdly, how do cognitive representations of covertly rehearsed skills change during MIP? Finally, what aspects of MI skills change over time as a function of MIP? Before addressing these issues, however, we must consider how MIP effects are typically explained.

## Explaining MIP Effects: Motor Simulation Theory

Motor simulation theory (MST; Jeannerod, 1994, 2001, 2006; see critique by O'Shea and Moran, 2017) offers an influential attempt to explain the efficacy of MIP. Briefly, this theory argues that imagined movement involves the internal simulation of actual movement. Three of its tenets may be summarized as follows. Firstly, actions are held to involve a covert stage during which they are simulated mentally. This covert stage includes the objective of the action and its consequences. Thus, it comprises “a representation of the future, which includes the goal of the action, the means to reach it, and its consequences on the organism and the external world” (Jeannerod, 2001, p. S103). Secondly, MST proposes that imagined and executed actions share a motor representation of an intention to act. Whereas, this intention is converted into an actual physical movement in the case of overt actions, it is not executed in the case of imagined actions. Thirdly, MST postulates that there is a functional equivalence between the simulation and execution of actions. The logic here is that since MI and executed actions are “assigned to the same motor representation vehicle” (Jeannerod, 1994, p. 190), then “motor imagery … should involve, in the subject's motor brain, neural mechanisms similar to those operating during the real action” (Jeannerod, 2001, pp. S103–S104). This latter proposition is the functional equivalence hypothesis (see review by Moran et al., 2012). It is supported by behavioral and neuropsychological evidence. For example, behavioral studies reveal a close correspondence between the durations of imagined and actual movements (see review by Guillot and Collet, 2005) and neuroimaging studies suggest that MI activates brain regions that partially overlap with those engaged in motor execution. These regions include the supplementary motor area (SMA), premotor cortex (PMC), primary motor cortex (M1), posterior parietal regions (e.g., the inferior and superior parietal lobes), the basal ganglia (BG) and cerebellum (see Hardwick et al., 2018). Furthermore, evidence demonstrates that MIP not only activates motor cortical and subcortical regions but also induces plastic change in motor networks and modulates synaptic activity at the spinal level (e.g., Debarnot et al., 2014; Grosprêtre et al., 2019). Interestingly, the latter effect indicates that motor commands are issued during MI and while they remain subthreshold for physical movement, they have the capacity to influence spinal cord activity (Grosprêtre et al., 2018, 2019). In summary, MST claims that imagined movements are functionally equivalent to executed ones with regard to intention, planning, and engagement of neural circuitry.

## Representing Procedural Instructions in Imagery Scripts

In the traditional MIP paradigm, participants in the cognitive rehearsal condition receive a scripted sequence of instructions designed to help them to see and feel the target skill in their imagination. Surprisingly, little is known about how people translate such verbal or written instructions into procedural skill representations. Nevertheless, sport psychology researchers like Holmes and Collins (2001) and Williams et al. (2013a) have developed useful, theoretically-based guidelines to improve MI scripts and interventions. Unfortunately, these guidelines do not address the processes by which participants create a mental representation of the instructions provided in imagery scripts. However, some insights into this latter issue come indirectly from a recent study by Theeuwes et al. (2018). Briefly, these authors developed a paradigm to study how MI affects the selection and retrieval of a novel response sequence. The paradigm involved a choice reaction time task in which participants respond to a picture by entering a designated response sequence. Results showed that MI led to an improvement in the application of novel instructions. However, this improvement appeared to be due more to enhanced response selection than to enhanced movement execution. Applying this finding to an MIP setting, it would be interesting to investigate how verbal instructions for a sporting skill (e.g., golf putting) are procedurally represented when the task has both perceptual (response selection) and motor (action execution) components. More generally, although the neural overlap between language and motor systems has been explored (e.g., see Pulvermüller, 2018), the supporting cognitive processes (e.g., the “translation” mechanism underlying verbal-to-procedural codes) remain unclear. The integration of semantic (verbal) and procedural (action) information may occur post-lexically via fast motor simulation (Frak et al., 2010). Alternatively, motor processing (i.e., simulation) may be inherent in action-related semantic processing (Pulvermüller, 2005). Perhaps instructions to respond to a stimulus automatically bias attention toward that stimulus (Tibboel et al., 2016). By association, scripted action components may bias attention toward the action itself. It should be noted, however, that individual differences and/or differences in processing strategy may also influence the interactive processing of procedural and semantic information. For example, research demonstrates that action context and experience influence the strength of association between semantic and procedural representations, with facilitation of linguistic processing being observed with greater action experience and where action components and linguistic content are congruently matched (Williams et al., 2013b; Beauprez et al., 2020). It would be interesting for future research to explore the effect of motor-related experience on the encoding of action instructions and whether or not MIP has a directional influence on this process.

## MIP and Working Memory (WM)

WM is a cognitive system that stores and manipulates currently relevant information for short periods of time (D'Esposito and Postle, 2015). Unfortunately, few studies have addressed its role in MI. This neglect is curious because during MI, action-related information is retrieved from long-term memory (LTM) and temporarily stored and manipulated in order to achieve intended action goals. In MI, action consequences must be maintained in mind (generated via simulation), control must be exerted over execution, and the action must be terminated (Jeannerod, 2006). So, how does MIP interact with WM?

In sport, expert performers are adept at prioritizing specific contextual and motor-relevant information in order to anticipate opponents' offensive plays (e.g., see Murphy et al., 2019). Clearly, expert athletes have developed elaborate mental representations that facilitate superior WM functioning. They also show superior WM span for visually presented movements compared to less skilled counterparts (Moreau, 2013). Interestingly, MIP appears to increase the neural efficiency of WM systems. For example, the dorsolateral prefrontal cortex (dlPFC) plays a crucial role in the maintenance and manipulation of information (Ptak, 2012) and is active during MI (Hardwick et al., 2018). Research shows that MIP elicits functional reorganization in neural systems with reduced activity in the dlPFC (WM center) signaling increased efficiency (Sauvage et al., 2015). However, because little research has explored the interaction between MI and memory systems, it is unclear if WM is actually the primary memory system supporting MI. Indeed, given recent neuroimaging evidence that mental imagery mediates the relationship between episodic information retrieval and divergent thinking (see Zhang et al., 2019), it may be possible that MIP influences how motor-related information is processed in WM. As we discuss in the next section, changes in mental representations occur following MIP (Frank et al., 2014). Also, LTM contributes to the formation of vivid/rich images in WM (Baddeley and Andrade, 2000). The capacity to form rich images is influenced by a host of factors such as (i) whether or not the imagery is dynamic in nature (and hence requires more information than if static; Baddeley and Andrade, 2000); (ii) whether or not the imagery contains unknown elements (as the amount of relevant information held in LTM will be limited); and (iii) the capacity of semantic memory (necessary to transform verbal cues into images; Baddeley and Andrade, 2000). Overall, given the link between MIP and memory, it seems important to investigate the relative role of WM compared with that of LTM in MIP and how MIP might influence these memory systems (e.g., Lauber et al., 2019).

## MIP and Changes in Mental Representation

A key tenet of modern cognitive psychology is the assumption that human behavior is causally related to “mental representations”—knowledge structures in memory that correspond to objects of thought. Accordingly, Schack and Ritter (2009) cognitive action architecture approach postulated that actions are represented in LTM as networks comprising hierarchically organized clusters of “basic action concepts.” These concepts “chunk” movements/body postures and their corresponding perceptual effects as tools to achieve specific action goals (Frank et al., 2016). Typically, they correspond to functionally meaningful elements of a given skill. Overall, the cognitive action architecture approach proposes that repeated execution of a skill—whether actual or imagined—produces modification of basic action concepts in LTM.

What effect does MIP have on mental representation development in skill learners? Frank et al. (2014) investigated the influence of MIP and physical practice on the development of mental representations in novice golfers. These novices were required to practice a golf putt mentally, physically, or in a combination of both modes of training over 3 days, while a control group did not practice at all. Participants' putting performance and mental representation structures were tested before and after the MIP intervention as well as after a 3 day retention interval. Results showed that participants who had practiced mentally, either solely or in combination with physical practice, developed mental representation structures that were more functional (i.e., were more similar to those of experts) than were those of participants who had not practiced mentally. Interestingly, Meier et al. (2020) showed recently that athletes' mental representations of the tennis serve changed over time as a function of coaching instructions (explicit or analogy-based). The findings of Frank et al. (2014, 2016) suggest that MIP operates at a relatively high level in the motor system—refining and elaborating athletes' mental representations of action during early stages of skill acquisition. Previously, Debarnot et al. (2014) concluded that expert and novice movement performance are distinguishable in terms of the neural substrates of relevant motor plans. Other studies suggest that the movement representations of experts capture more faithfully the biomechanical components of domain-specific movement than do those of novices (for review see Bläsing et al., 2012). However, even though behavioral changes have been observed following MIP (Debarnot et al., 2014), the extent and pattern of representational change following MIP (without physical practice) have yet to be fully elucidated—particularly in expert populations (for an examination of representational change over the course of a MIP program in novice sports performers, see Kim et al., 2017).

## MIP and Changes in MI Ability

There is evidence to suggest that MIP can improve MI ability (Rodgers et al., 1991; Ruffino et al., 2017). For example, Rodgers et al. (1991) reported that a 16-week figure skating imagery training program enhanced figure skaters' ability to imagine basic movements. This finding has been replicated in other sporting contexts such as golf (Williams et al., 2013b) and synchronized skating (Cumming and Ste-Marie, 2001). More recently, Mateo et al. (2018) reported that a 5 week MI training program for people with quadriplegia improved self-rated aspects of their imagery experience (namely, its vividness). Unfortunately, this study was descriptive rather than experimental in nature and did not use a psychometric measure of MI ability both before and after MI training. Interestingly, subjective indices of imagery appear to have a distinctive neural signature. For example, Zabicki et al. (2019) discovered that reports of imagery vividness are linked to consistent patterns of neural activity in premotor cortical areas, which may reflect kinesthetic retrieval of motor representations. Clearly, a systematic program of theory-driven research is required to identify which specific components of MI are affected by MIP. To facilitate such a study, a longitudinal research design is required in which data are collected using a combination of psychometric, chronometric, and psychophysiological measures of MI (see Collet et al., 2011; Williams et al., 2015).

## Conclusions

Although at first glance, thinking and action appear to lie at opposite ends of the behavioral spectrum, cognition and action are elaborately interconnected (Rosenbaum et al., 2012)—as is evident in our discussion of the theoretical foundations of MI. According to MST, MI activates neural motor systems via a simulation mechanism that is supported and supervised by higher cognitive systems (Jeannerod, 2006). Unfortunately, as this opinion paper has highlighted, many of the cognitive systems implicated by, and/or associated with, MIP remain unclear.

## Author Contributions

All authors listed have made a substantial, direct and intellectual contribution to the work, and approved it for publication.

### Conflict of Interest

The authors declare that the research was conducted in the absence of any commercial or financial relationships that could be construed as a potential conflict of interest.
